# Evaluation of lactulose, lactose, and fructose breath testing in clinical practice: A focus on methane

**DOI:** 10.1002/jgh3.12240

**Published:** 2019-08-20

**Authors:** Ruth M Harvie, Caroline J Tuck, Michael Schultz

**Affiliations:** ^1^ Department of Medicine, Dunedin School of Medicine University of Otago Dunedin New Zealand; ^2^ Gastrointestinal Disease Research Unit, Kingston General Hospital Queen's University Kingston Ontario Canada; ^3^ Gastroenterology Otago Ltd., Marinoto Clinic Mercy Hospital Dunedin New Zealand

**Keywords:** breath tests, irritable bowel syndrome, methane, sensitivity and specificity

## Abstract

**Background and Aim:**

Breath testing (BT) is used to identify carbohydrate malabsorption and small intestine bacterial overgrowth. Measuring methane alongside hydrogen is advocated to reduce false‐negative studies, but the variability of methane production is unknown. The aim of this study is to examine the effect of high methane production on hydrogen excretion after ingesting lactulose, fructose, or lactose.

**Methods:**

A retrospective audit was performed of patients with gastrointestinal symptoms who underwent BT. Following a low fermentable carbohydrate diet for 24‐h, a fasting BT before consuming 35 ml lactulose, 35 g fructose, or lactose in 200 ml water, followed by BT every 10–15 min for up to 3‐h, was performed. A positive test was defined as a ≥20 ppm rise of hydrogen or methane from baseline. A high methane producer had an initial reading of ≥5 ppm. Breath hydrogen and methane production were measured as area under the curve. Chi‐squared tests were used to compare proportions of those meeting the cut‐off criteria.

**Results:**

Of patients, 26% (28/106) were high methane producers at their initial lactulose test. The test–retest repeatability of methane production was high, with the same methane production status before ingesting lactose in all (70/70) and before ingesting fructose in most (71/73). Methane production was highly variable during testing, with 38% (10/26) having ≥1 reading lower than baseline. Hydrogen produced by high or low methane producers did not differ (1528 [960–3645] ppm min *vs* 2375 [1810–3195] ppm min [*P* = 0.11]). Symptoms and breath test results were not positively related.

**Conclusion:**

The validity of including an increase of ≥20 ppm methane to identify carbohydrate malabsorption or small intestine bacterial overgrowth should be questioned due to the variability of readings during testing.

## Graphical abstract

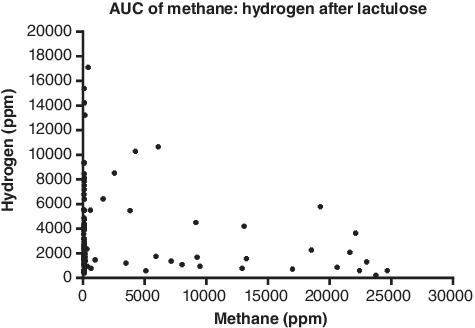

Breath testing is used to identify carbohydrate malabsorption and small intestine bacterial overgrowth. Measuring methane alongside hydrogen is advocated to reduce false‐negative studies, but the variability of methane production is unknown. The validity of including an increase of ≥20 ppm methane to identify carbohydrate malabsorption or small intestine bacterial overgrowth should be questioned due to the variability of readings during testing.

## Introduction

Irritable bowel syndrome (IBS) is a common gastrointestinal condition that affects ~11% of the population.^1^ It is heterogeneous in both presentation and pathophysiology, with an altered brain–gut axis, dysbiosis, impaired gastrointestinal transit, altered immune function, bile acid malabsorption, psychological distress,^2^ and—more controversially—small intestine bacterial overgrowth (SIBO),^3^ all possibly contributing to its development. Due to the lack of defined pathophysiology, treatment has been suboptimal. Furthermore, there are no established biomarkers in IBS,^4^ either to identify it as an entity or to inform treatment strategies.

Hydrogen breath testing after the administration of a carbohydrate substrate such as fructose, lactose, or lactulose is used to identify carbohydrate malabsorption^5^, ^6^ or SIBO.^7^ The principle of breath testing is that substrates escaping digestion undergo fermentation by microorganisms, causing gas release, which is then excreted by the lungs and can be measured.^8^ Lactose and small amounts of fructose should be absorbed in the small intestine under normal physiological conditions, and any rise in hydrogen production should be due to malabsorption of fructose or lactose.^8^ Lactulose is not absorbed in the small intestine as humans lack the enzymes required to break it down,^9^ and an early rise in hydrogen following lactulose is believed to indicate SIBO.^3^ An alternative substrate used to identify SIBO is glucose, thought to indicate proximal small intestinal bacterial fermentation if breath testing is positive as it is normally well absorbed. However, the use of breath testing for identifying SIBO is controversial.^10^, ^11^

A lack of standardized protocols has hampered the use of breath testing for the identification of SIBO and carbohydrate malabsorption and, subsequently, for directing treatment.^12^, ^13^ The lack of standardization of tests extends to the choice of substrate for identifying SIBO (either lactulose or glucose), as well as differences in cut‐off values used to define malabsorption, test duration, and substrate dose. This has led to vast differences in the proportion of patients identified with SIBO by substrate used. When glucose is used, the proportion is 31% (95% confidence interval [CI], 14–50), and when lactulose is used, the proportion is 54% (95% CI, 32–76)^.^ 14 The most commonly used machines for analyzing breath samples can now measure breath hydrogen and methane simultaneously, and testing now routinely includes measurement of methane. Because it takes 4 mol of hydrogen to produce 1 mol of methane by bacterial fermentation, many believe it is important to measure methane levels as high methane producers may produce less hydrogen.^15^

It has been suggested that breath tests can be used to direct therapy, including dietary therapy, and the implementation of a low fermentable oligosaccharide, disaccharide, monosaccharide, and polyol diet.^16^ In particular, patients have been encouraged to limit the number of foods that are restricted as lactose is the disaccharide and fructose is the monosaccharide that may need to be restricted during the diet. Therefore, if there is no rise in breath hydrogen or methane levels, these foods may not need to be restricted. Moreover, the amount of substrate used in the tests is much larger than typically consumed in the diet and might not lead to physiologically acceptable results.^17^

In this paper, results of breath testing using lactulose, fructose, and lactose as the substrates in a clinical, real‐world population will be examined, with particular attention given to the role of methane and whether including a rise of ≥20 ppm methane from the initial reading is valuable in identifying SIBO and carbohydrate malabsorption.

## Methods

### *Patients*

This study was a retrospective clinical audit of patients who were referred for breath testing from July 2014 to June 2017 due to the presence of gastrointestinal symptoms compatible with IBS. Patients were referred for the identification of either SIBO by lactulose or malabsorption of fructose or lactose. The study was approved by the University of Otago Human Ethics Committee (Health) (HE15/008).

Clinical data were extracted from paper records located at Gastroenterology Otago Ltd. and the electronic record at the Southern District Health Board.

### *Breath testing*

Breath testing was performed on three separate days, with at least 1 day in between for each of lactulose, lactose, and fructose. Restrictions before the test were as follows: avoid antibiotics and probiotics for 2 weeks, follow a diet low in fermentable carbohydrates for 24 h before the test, fast overnight, and avoid smoking and using perfume for 10 h before testing. Test technicians confirmed compliance with the pretest diet prior to test commencement.

All breath samples were analyzed for hydrogen and methane levels by gas chromatography on a Bedfont GastroCH_4_ECK™ (Bedfont Scientific Ltd., Kent, UK). After an overnight fast, patients consumed 35 ml of lactulose, or 35 g fructose, or 35 g lactose dissolved in 200 ml water. Before ingesting the test substrate, all patients provided an initial breath sample and a sample every 10 min thereafter for 180 min for lactulose or every 15 min for 180 min for fructose and lactose or until they were positively identified with SIBO or a carbohydrate malabsorption. The cut‐off value for identifying SIBO was an increase of ≥20 ppm from the initial reading within 90 min of the administration of lactulose for either hydrogen or methane or both. The cut‐off value for identifying fructose and lactose malabsorption was ≥20 ppm from the initial reading. A high methane producer was defined as someone with an initial reading of ≥5 ppm of methane, and a low methane producer had an initial reading of <4 ppm of methane.

Patients recorded their own breath test measurements and noted any symptoms they experienced during the test using a standardized form whereby symptoms at each breath sample could be recorded if they occurred. Breath hydrogen and methane production were quantitatively assessed by measuring the area under the curve (AUC) for the duration of the test. To test for variation within methane breath tests, values were normalized to zero, and the number of fluctuations below the initial reading was compared to the rise above the initial reading.

### *Patient assessments*

At their first breath test, patients completed a gastrointestinal symptom questionnaire rating the severity of 20 GI symptoms that they had experienced within the week before testing (1–7 points with increasing severity). These symptoms included pain, bloating, diarrhea, constipation, early satiety, and nausea.^18^, ^19^, ^20^

### *Statistical analysis*

Data were analyzed using STATA IC13 (StataCorp, College Station, ATX, USA), with graphs drawn in GraphPad Prism version 7.03 (GraphPad Software, San Diego, CA, USA). Descriptive data are reported as mean and standard deviation or median and 95% confidence interval as indicated. Chi‐squared tests were used to compare proportions of those identified with SIBO or a carbohydrate malabsorption. Student's t‐tests were used to compare characteristics if the data were normally distributed; otherwise, Mann–Whitney *U*‐tests were used.

## Results

There were 106 patients who underwent lactulose breath testing, of whom 70 underwent a lactose breath test, and 73 underwent a fructose breath test. Patients were 42.5 ± 16.2 years old, and 75% (79/106) of patients were female. All patients had IBS or IBS‐like symptoms. Gastrointestinal comorbidities experienced by patients were gastroesophageal reflux (four), coeliac disease (four), Crohn's disease (five), and hemorrhoids (four). Two patients had had a colectomy. Other medical comorbidities included asthma (six), arthritis (four), eczema (three), depression (two) anxiety or stress (five), allergic rhinitis (three), and previous eating disorder (three).

A total of 71% (75/106) of patients were identified to have SIBO by either hydrogen or methane on lactulose breath test. Symptoms were reported in 42% (45/106) of patients during the lactulose breath test (Table 1). Patients reporting symptoms during testing with lactulose were less likely than patients who did not report symptoms to have a rise in gas levels: 60% (27/45) *versus* 79% (48/61) (*P* < 0.05).

**Table 1:** Proportion of patients diagnosed with small intestine bacterial overgrowth or fructose or lactose malabsorption by a rise in breath hydrogen

	Lactulose (*n* = 106), *n* (%)	Fructose (*n* = 73), *n* (%)	Lactose (*n* = 70), *n* (%)
Diagnosed with malabsorption			
Neither gas	31 (29)	47 (64)	56 (80)
Hydrogen only	58 (55)	22 (30)	11(15)
Methane only	10 (9)	5 (7)	3 (4)
Both gases	7 (7)	0 (0)	0 (0)
Total number diagnosed	75 (71)	27 (37)	14 (20)
Any symptom	45 (42)	25 (34)	21 (30)
Pain	12 (11)	5 (7)	1 (1)
Borborygmi	18 (17)	4 (5)	2 (3)
Cramping	6 (6)	3 (4)	1 (1)
Diarrhea	7 (7)	7 (4)	2 (3)
Headache	7 (7)	2 (3)	4 (6)
Nausea	17 (16)	9 (12)	2 (3)
Flatulence	11 (10)	3 (4)	3 (4)
Lightheaded/brain fog	7 (7)	0 (0)	0 (0)
Bloated	11(10)	5 (7)	5 (7)

Patients ingested 35 mL lactulose, and small intestine bacterial overgrowth was diagnosed by an increase in ≥20 ppm from baseline within 90 min. A total of 35 g of fructose or 35 g lactose dissolved in 200 mL water was used as the substrate for fructose and lactose malabsorption, with a positive test being ≥20 ppm above baseline. Breath hydrogen and methane levels were measured by gas chromatography on a GastroCH_4_ECK (Bedfont Scientific Ltd., Kent, UK).

There were 16% (11/70) of patients with lactose malabsorption identified by a rise in breath hydrogen, with an additional three people identified through elevated methane levels (Table 1). There was no relationship between symptoms and a rise in either breath hydrogen (*P* = 0.53) or methane during lactose testing (*P* = 0.85).

There were 30% (22/73) of patients who were positive for fructose malabsorption by raised hydrogen levels and five by elevated methane levels (Table 1). During fructose testing, those who reported symptoms were less likely than those who did not report symptoms to demonstrate a rise in either or both gases: 27% (13/48) *versus* 56% (14/25) (*P* < 0.05).

### *Repeatability of baseline methane levels*

Of the participants, 26% (28/106) were high methane producers on their initial lactulose test, and 21% (15/70) and 21% (15/73) produced high methane readings on their subsequent baseline lactose and fructose tests, respectively. The test–retest repeatability of methane production status was high, with the same methane production status at the baseline test before ingesting lactose in all patients (70/70) and before ingesting fructose in most (71/73). There was no difference in mean age of high (45.1 ± 16.5) *versus* low (41.7 ± 16.5) methane producers (*P* = 0.39) or the proportion who were female (Table 2).

**Table 2:** Differences in participants by methane production status

	High methane producer (28)	Low methane producer (78)	Significance
Age (years)	43.7 ± 16.8	42.0 ± 16.1	0.68
Gender, *n* (%)			
Female	17 (61)	62 (79)	
Male	11 (39)	16 (21)	0.05
Proportion with a significant rise in hydrogen in 90 min, *n* (%)			
Yes	17 (61)	64 (82)	
No	11 (39)	14 (18)	<0.05
Time to a rise in H_2_ (minutes)	60	65	0.84
95% confidence interval	50–90	60–80	
Amount of H_2_ produced (area under the curve: ppm min)	1528	2375	0.11
	960–3645	1810–3195	

Breath hydrogen and breath methane levels were measured by gas chromatography on a GastroCH_4_ECK (Bedfont Scientific Ltd., Kent, UK). Mann–Whitney *U*‐tests were used to test for statistical significance.

### *Symptom questionnaire*

**Figure 1:**
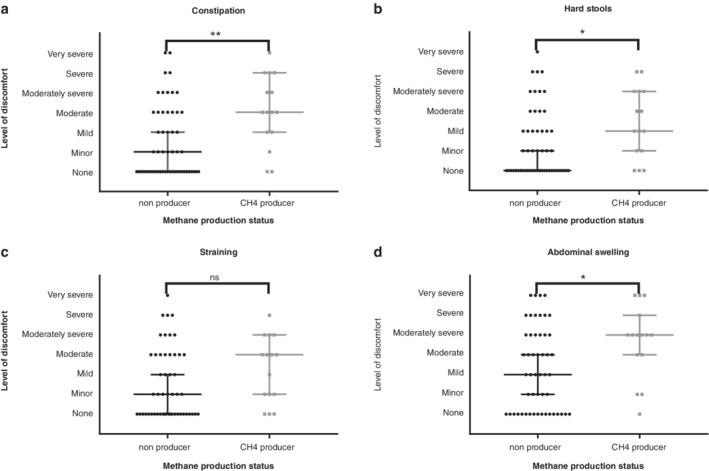
Severity of symptoms by whether patients produced breath methane. A methane producer was defined as having an initial methane reading of ≥5 ppm. Methane levels were measured by gas chromatography on a GastroCH4ECK. Patients completed a gastrointestinal symptom questionnaire (18) using a 7‐point Likert scale. *P < 0.05; **P < 0.01.

Seventy patients completed the symptom questionnaire at baseline. Whether participants did or did not have a >20 ppm rise in breath hydrogen reading from their initial reading was not associated with any differences on the symptom questionnaire. Being a high methane producer was associated with more severe constipation (*P* < 0.01) (Fig. 1a) and hard stools (*P* < 0.05) (Fig. 1b) but not straining (*P* = 0.08) (Fig. 1c). High methane producers were also more likely to have worse abdominal swelling than low producers (*P* < 0.05) (Fig. 1d). None of the other symptoms showed statistical significance.

### *Hydrogen testing after lactulose ingestion*

There were only two patients who did not have a reading of >5 ppm of hydrogen during testing. Both of these patients produced very high levels of methane throughout their testing. There were some fluctuations during testing, with 40 patients having at least one reading lower than their initial reading and 12 patients having at least one reading >3 ppm lower than their initial reading. There were only six patients who had at least one reading >3 ppm lower than their initial reading and a reading at least 20 ppm higher than their initial reading. For five of the six, the reading with the largest difference was 4–7 ppm lower than their initial reading, and the final patient had an initial reading of 29 ppm. The reading that had the largest drop from the initial reading was 14 ppm.

### *Methane testing after lactulose ingestion*

**Figure 2:**
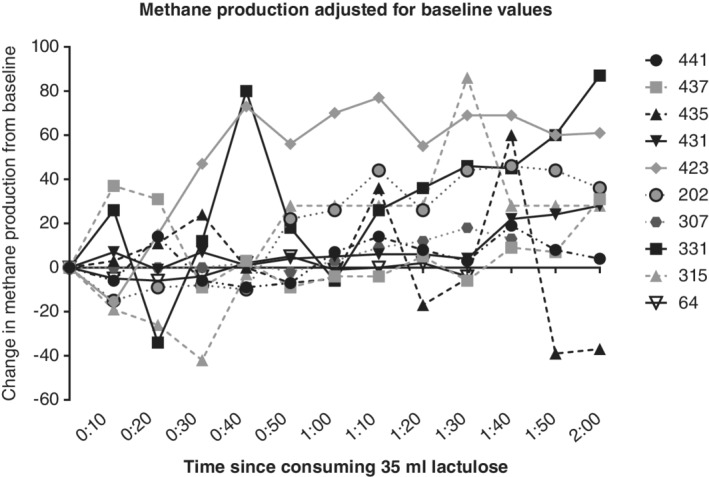
Change in breath methane production from initial reading after consuming 35 mL of lactulose in patients who had at least one level lower than their initial reading. Levels are normalized to a starting value of 0 to more clearly show that methane levels for some people dropped below their initial value. Breath methane levels were measured by gas chromatography on a GastroCH4ECK (Bedfont Scientific Ltd., Kent, UK). Patient IDs are indicated on the right‐hand side of the graph.

**Figure 3:**
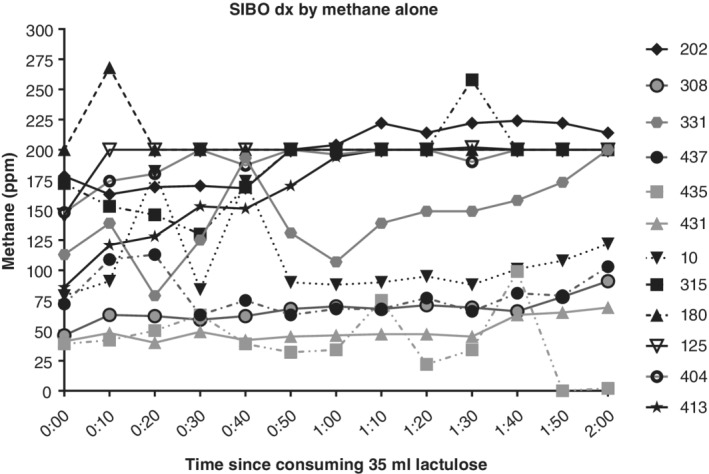
Breath methane levels measured before consuming 35 mL of lactulose solution and every 10 min thereafter for 2 h. Breath methane levels were measured by gas chromatography on a GastroCH4ECK (Bedfont Scientific Ltd., Kent, UK).

There were only two patients who had an initial reading <5 on lactulose testing who had any methane readings above 5 ppm during lactulose testing, and these two patients had an initial reading of 4 ppm, which rose to 6 ppm. For high methane producers, their methane readings fluctuated throughout all three tests. Of all high methane producers, 39% (11/28), including patients who had a concurrent rise in hydrogen, had at least one methane reading lower than the initial reading (Fig. 2); 11% (12/106) were identified with SIBO based solely on an increase in methane (Fig. 3). Of these 12 patients, 50% (6/12) had ≥1 reading below their initial reading (Fig. 3).

**Figure 4:**
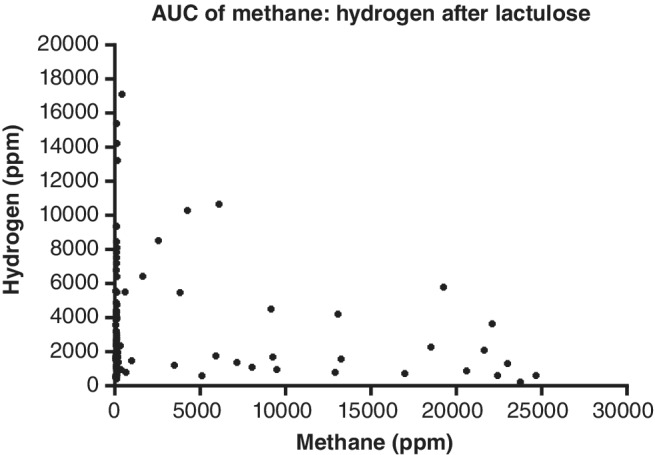
Area under the curve (AUC) of methane (ppm min) produced during 2‐h testing compared to the AUC of hydrogen (ppm min) after consuming 35 mL lactulose. Breath hydrogen and methane levels were measured by gas chromatography on a GastroCH4ECK (Bedfont Scientific Ltd., Kent, UK).

There were 61% (17/28) high methane producers who had a concurrent rise in hydrogen of ≥20 ppm from their initial reading. Of high methane producers, 18% (5/28) had a significant rise in hydrogen but did not have a significant rise in methane. Compared to patients who were low methane producers, there was a lower proportion of high methane producers with a ≥ 20 ppm rise in breath hydrogen levels: 61% (17/28) *versus* 82% (64/78) (*P* < 0.05) (Table 2). Otherwise, being a high methane producer did not affect other markers of hydrogen production. There was no difference in the time to a ≥ 20 ppm rise in hydrogen between high methane producers (70 min, 95% CI 60–80) and low methane producers (60 min, 95% CI 50–90) (*P* = 0.10). There was no difference in the total amount of hydrogen produced, as measured by the AUC, by level of methane production (Table 2). Elevated methane AUCs were not associated with reduced hydrogen AUCs (Fig. 4). A total of 85% (22/26) of methane producers had a rise of ≥20 ppm in methane during the 2‐hour testing period. The four patients who did not have a significant rise in methane had an initial reading of <30 ppm.

### *Methane production during fructose and lactose breath testing*

**Figure 5:**
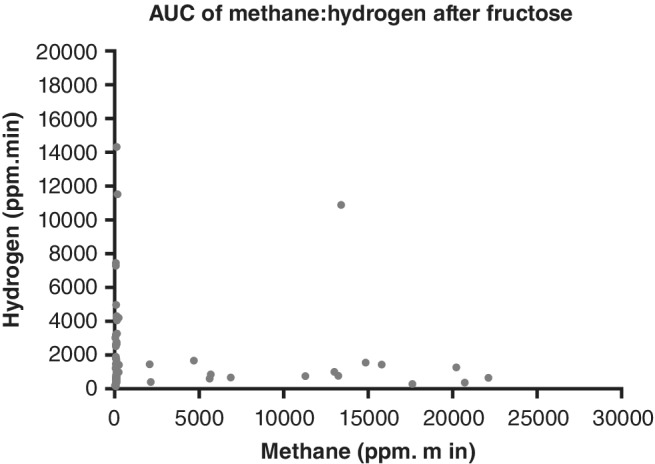
Area under the curve (AUC) of methane produced during 2‐h testing compared to the AUC of hydrogen after patients consumed 35 g of fructose dissolved in 200 mL water. Breath hydrogen and methane levels were measured by gas chromatography on a GastroCH4ECK (Bedfont Scientific Ltd., Kent, UK).

A total of 74 patients had fructose breath testing, with 21% (15/74) being high methane producers (Table 1). Methane production appeared to suppress hydrogen production, with all but one methane producer having a hydrogen AUC of <2000 ppm min (Fig. 5). However, there was no statistically significant difference between the total amount of hydrogen excreted by methane producers (865 ppm min, 95% CI 660–1455) compared to nonproducers (1091 ppm min, 95% CI 694–2681) (*P* = 0.33).

Of the high methane producers on initial reading, 27% (4/15) of methane producers had a rise of ≥20 ppm of methane, and 7% (1/15) had a significant rise in hydrogen following fructose ingestion. Methane levels during breath testing fluctuated, with 47% (7/15) patients having at least one reading below their initial reading.

Seventy patients had lactose breath testing, with 21% (15/70) being high methane producers on their initial test. Methane production appeared to suppress hydrogen production, with all high methane producers having <2000 ppm min of hydrogen in 90 min. However, there was no statistically significant difference in the total amount of hydrogen excreted between high (517.5 ppm min, 95% CI 300–1275) and low methane producers (750 ppm min, 95% CI 600–1088) (*P* = 0.10). Nearly three‐quarters of the high methane producers (73%, 11/15) had at least one methane level below their initial reading. There were only 2 of 15 high methane producers who had a rise of ≥20 ppm above their initial hydrogen level.

## Discussion

This audit raises questions about the inclusion of methane testing alongside hydrogen breath testing when assessing carbohydrate malabsorption and SIBO. Plotting of all methane results over time showed that methane production is highly variable, whereas hydrogen production was less variable, thus calling into question the validity of using an increase of ≥20 ppm methane from the initial reading as a criterion for identifying either SIBO or carbohydrate malabsorption. These fluctuations in breath methane levels increase the randomness of identification with SIBO or a carbohydrate malabsorption. Second, there was high repeatability between tests on methane production status if a level of ≥5 ppm was used to identify a methane producer. Finally, there was no relationship between reporting symptoms during testing and having a ≥ 20 ppm rise in either breath hydrogen or methane.

In this audit of 106 patients who underwent combined testing of methane and hydrogen, it was discovered that being a high methane producer did not suppress hydrogen production when lactulose was the substrate. This finding, if replicated in another larger cohort, is significant as the rationale for inclusion of methane analysis alongside hydrogen analysis when testing for SIBO or carbohydrate malabsorption is that hydrogen is consumed by archaea, which utilize 4 mol of hydrogen to produce 1 mol of methane. Therefore, when methane is produced, there is likely to be a reduction in hydrogen produced, and including methane in the test will improve its sensitivity.^15^, ^21^, ^22^

While those advocating for the inclusion of methane analysis in the breath test state its value in increasing sensitivity, less consideration has been given to its effect on test specificity, which will likely decrease. In this audit, the variability in methane readings if lactulose was used as a substrate makes it questionable whether a rise in breath methane levels is truly representative of the increased fermentation of the substrate provided or if the rise is independent of ingestion of lactulose. Methane is less subject to diet‐induced changes in levels than hydrogen,^23^ and previous work has shown methane excretion during fasting^.^ 24 There is poor reproducibility of the amount of methane produced during lactulose breath testing when the average methane AUC is different from zero.^25^ Poor repeatability of methane AUCs has been demonstrated when fructose is used as the substrate.^26^ Including a significant rise in methane in the criteria for identifying SIBO or carbohydrate malabsorption may increase false‐positive results and decrease test specificity. Increasing the number of false positives has important implications as the most commonly used algorithm recommends that those identified with SIBO be prescribed antibiotics, particularly rifaximin.^27^ Rifaximin is synthetic and not systemically absorbed; it is expensive, and often, repeated doses are required. Furthermore, the overuse of antibiotics can lead to antibiotic resistance^.^ 28

This audit validated doing a spot methane test using a cut‐off value of ≥5 ppm to identify high methane producers without the need to conduct the full 2–3‐hour breath testing regimen.^29^ In this audit, those with initial readings of <5 ppm never had a significant rise in methane levels that would trigger the identification of malabsorption, and only two patients were assigned a different methane production status across the three tests. In this case, spot methane studies could be used to identify methane production as a potential cause of constipation or to reduce the need to include methane analysis in studies of nonmethane producers.

Previous research has called into question other aspects of breath testing as the coadministration of lactulose with radio‐opaque markers showed that a rise in breath hydrogen usually occurred when the markers arrived at the ileocecal junction,^30^, ^31^ indicating that the early rise in breath hydrogen was due to the arrival of lactulose in the colon and fermentation by colonic bacteria. Time of arrival at the ileocecal junction varied considerably amongst participants, and in 88% of participants, the rise in hydrogen was after the radio‐opaque markers arrived in the ileocecal junction.^30^ Thus, the authors concluded that an early rise in hydrogen indicates rapid small bowel transit rather than SIBO. Furthermore, poor test–retest reproducibility of breath testing has been shown to have no correlation between hydrogen AUCs between tests,^32^ nor was there any correlation in time of the first rise in breath hydrogen levels between the two tests (*r* = 0.14, *P* = 0.54). Moreover, breath methane excretion was shown not to be an accurate marker of methane production compared to methane measured in rectal samples.^33^

The variability in methane levels during breath testing needs to be investigated in a larger cohort to substantiate results in this study, especially as it is known that breath methane levels fluctuate independent of dietary intake.^23^, ^24^ Due to the small sample size and because this was not a predetermined research question, our findings could have been a result of chance. Furthermore, no statistical model was developed to test whether the hydrogen to methane AUCs fit into the model that has been proposed for high breath methane excretion suppressing hydrogen production. Intraindividual variation of methane producers using the same substrate of the AUC for hydrogen and methane should be plotted using Bland and Altman statistics to test the reproducibility of results. This study used a variety or statistical and graphical methods to examine the effect of breath methane excretion on hydrogen production, and including the raw data in graphical form allows the reader to examine the data for themselves.

In conclusion, this audit suggests that identifying either carbohydrate malabsorption or SIBO by an increase of ≥20 ppm in methane producers needs to be questioned due to the variability in readings throughout testing. However, using a cut‐off value of ≥5 ppm of methane on a single time point breath test seems to identify methane producers.
